# Identification of nonessential regions of the nsp2 protein of an attenuated vaccine strain (HuN4-F112) of highly pathogenic porcine reproductive and respiratory syndrome virus for replication in marc-145 cell

**DOI:** 10.1186/1743-422X-9-141

**Published:** 2012-08-02

**Authors:** Yan-Zhao Xu, Yan-Jun Zhou, Shan-Rui Zhang, Wu Tong, Ling Li, Yi-Feng Jiang, Guang-Zhi Tong

**Affiliations:** 1 Division of Swine Infectious Diseases, Shanghai Veterinary Research Institute, Chinese Academy of Agricultural Sciences, No.518, Ziyue Road, Minhang District, Shanghai, 200241, China

**Keywords:** HuN4-F112, Nsp2, Nonessential region, Deletion mutant

## Abstract

**Background:**

The regions in the middle of nonstructural protein 2 (nsp2) of porcine reproductive and respiratory syndrome virus (PRRSV) have been shown to be nonessential for PRRSV replication, and these nonessential regions are different in various viral strains.

**Finding:**

In this study, the nonessential regions of the nsp2 of an attenuated vaccine strain (HuN4-F112) of highly pathogenic porcine reproductive and respiratory syndrome virus were identified based on an infectious cDNA clone of HuN4-F112. The results demonstrated that the segments of nsp2 [amino acids (aa) 480 to 667] tolerated deletions. Characterization of the mutants demonstrated that those with small deletions did not affect the viral growth on Marc-145 cells, but deletion of these regions led to earlier PRRSV replication increased (before 36 h after infectious *in vitro*).

**Conclusion:**

The functional roles of nsp2 variable middle region for PRRSV HuN4-F112 replication have been identified. Our results also suggested that none-essential region might be an ideal insertion region to express foreign gene in PRRSV genome.

## Finding

Porcine reproductive and respiratory syndrome virus (PRRSV) is an enveloped, positive-stranded RNA virus belonging to the family *Arteriviridae*[1]. Based on a difference in nucleotide sequence, two PRRSV genotypes have been identified including European genotype (type 1) and North American genotype (type 2) represented by prototype viruses Lelystad and VR-2332, respectively [2-5]. The genome of PRRSV is approximately 15 kb in size, and it contains ten open reading frames (ORF), designated as ORF1a, ORF1b, ORF2a, ORF2b, and ORF 3 to ORF5, ORF5a, ORF6 and ORF7 [3,6-10]. ORF1a and ORF1b encode viral nonstructural proteins which are directly translated upon viral entry. Then the polyproteins are cleaved into 14 nonstructural protein (nsp) (nsp1α, nsp1β, nsp2 to nsp6, nsp7α, nsp7β, and nsp8 to nsp12) that participate viral replication and transcription. Among the 14 nonstructural proteins, the nsp2 protein is the largest PRRSV replicative protein [2,11,12]. Through the alignment of arterivirus nsp2 proteins, nsp2 could be recognized containing three major domains: a N-terminal cysteine proteinase domain (PL2), a middle region, and a hydrophobic transmembrane (TM) region close to the C terminus [11,13-15]. Nsp2 has been shown to be highly heterogeneous and variable, and become a key factor in distinguishing between PRRSV type 1 and type 2 strains due to their length difference and less than 40% similarity of their amino acid sequences [2,4,16].

It was reported that natural mutations, insertions, or deletions always occurred in the middle region or near to the N-terminal region of the nsp2, while the putative PL2 domain and TM domain remains well conserved [2,4,13,17-22]. In 2006, a highly pathogenic porcine reproductive and respiratory syndrome virus (HP-PRRSV) (a virulent form of PRRSV, i.e., HP-PRRSV HuN4) was identified in China [21,23,24]. HuN4-F112 was an attenuated strain by passaging the HP-PRRSV HuN4 on Marc-145 cell in our laboratory [25]. One character of the virulent PRRSV strain was two discontinuous deletions of 1 aa and 29 aa at positions 482 and 534–562, respectively, relative VR-2332 [21,26]. So in our study, we wanted to explore the genetic flexibility of the virulent form of PRRSV nsp2 protein and identified the nonessential regions in the middle of nsp2 of HP-PRRSV Strain HuN4-F112.

**Figure 1:**
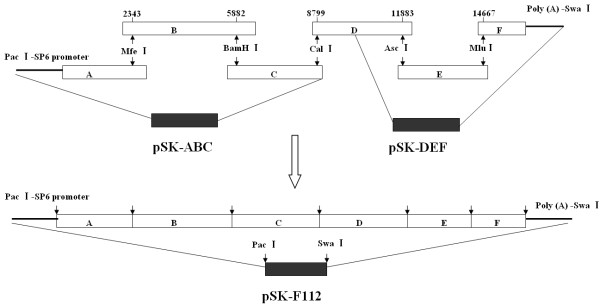
**Assembly of the full-length cDNA clone of PRRSV strain HuN4-F112.** The total genome was amplified in six fragments **(A to E)**. Each of these fragments was cloned to pSK vector with unique restriction enzyme cleavage site, respectively. Then, **A-B-C** and **D-E-F** were linked into pSK, respectively. Finally, the full-length clone was assembled from pSK-ABC and pSK-DEF with *Pac*I and *Cla*I . A SP6 polymerase promoter and genetic marker (*Mlu*I enzyme cleavage sites at 14667nt) were added by PCR. Arrowheads and numbers indicate enzyme cleavage sites in PRRSV strain HuN4-F112.

Full-length cDNA clone of HuN4-F112 was constructed as shown in Figure 1 as according to Zhang *et al.*[27]. The cDNA clone (pSK-F112) contains the SP6 RNA polymerase promoter at the 5′-end of PRRSV genome, the 3′-end sequence flanked the 32 poly (A), and a *Mlu*I restriction enzyme site at nt 14667 which was a genetic marker to differentiate the rescued viruses and parental virus (HuN4-F112).

**Figure 2:**
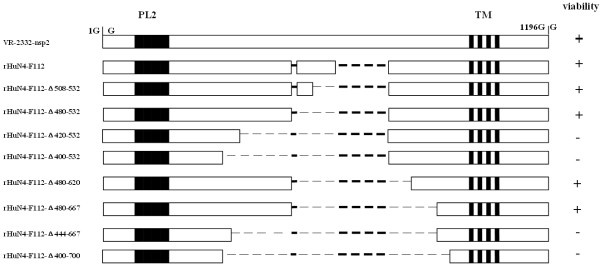
**Construction of PRRSV strain HuN4-F112 nsp2 mutants.** Mutagenesis PCR was used to delete and insert fragment in the nsp2 region. The deleted amino acids are shown as thin dashed lines, thick dashed lines indicate the sequence naturally deleted as compared with the North American genotype representative virus VR-2332. The putative enzyme domain (PL2) and TM region are indicated in the figure. The viability for each mutant constructed is shown on the right: +, viable; -, nonviable.

The nsp2 deletion strains were generated by the Quick Change Multi site-directed mutagenesis kit (Stratagene) according to the manufacturer’s recommendations. The deletion regions of nsp2 were showed in Figure 2. Sequences of deletion primers were listed in Table 1. The construction of the deletion full-length cDNA clone was performed in two steps. First, the deletion fragments ABC (named pSK-ABC-Δ) were created by mutagenesis kit. Then, the deletion full-length infectious clones were assembled by ligating fragments DEF with pSK-ABC-Δ as shown on Figure 1.

**Table 1:** Oligonucleotide primers used in this study

***primer***	***Sequence***
FΔ480-532	5′- TCCCTAACGGTTCGGAAGAAACAACGCTGACGCACCAGGA -3′
RΔ480-532	5′- TCCTGGTGCGTCAGCGTTGTTTCTTCCGAACCGTTAGGGA -3′
FΔ508-532	5′- AGCCCGTACTTATGCCCGCGACAACGCTGACGCACCAGGA -3′
RΔ508-532	5′- TCCTGGTGCGTCAGCGTTGTCGCGGGCATAAGTACGGGCT -3′
FΔ480-620	5′-TCCCTAACGGTTCGGAAGAAGTGTCATCAAGCAGCCCCCT-3
RΔ480-620	5′-AGGGGGCTGCTTGATGACACTTCTTCCGAACCGTTAGGGA-3
FΔ480-667	5′-TCCCTAACGGTTCGGAAGAATGTGATGCGTCCAAGCTTGG-3′
RΔ480-667	5′-CCAAGCTTGGACGCATCACATTCTTCCGAACCGTTAGGGA-3′
FΔ444-667	5′-CTCGAAGAACAAAGTCTGTCTGTGATGCGTCCAAGCTTGG-3′
RΔ444-667	5′-CCAAGCTTGGACGCATCACAGACAGACTTTGTTCTTCGAG-3′
FΔ420-532	5′-AGCAGGTCAATTTAAAAGCTACAACGCTGACGCACCAGGA-3′
RΔ420-532	5′-TCCTGGTGCGTCAGCGTTGTAGCTTTTAAATTGACCTGCT-3′
FΔ400-532	5′-AGGATCTGCTAAAACTAGCCACAACGCTGACGCACCAGGA-3′
RΔ400-532	5′-TCCTGGTGCGTCAGCGTTGTGGCTAGTTTTAGCAGATCCT-3′
FΔ400-700	5′-AGGATCTGCTAAAACTAGCCCAGGCGTTTCGCATCTTAAG-3′
RΔ400-700	5′-CTTAAGATGCGAAACGCCTGGGCTAGTTTTAGCAGATCCT-3′
F2436	5′-ATATGGGCTCATGTCCACTG- 3′
R3442	5′-GTAACATCACAAACCCGCAC- 3′
F14320	5′- CACAGCTCCACAGAAGGTGC-3′
R14752	5′- TAACAGCTTTTCTGCCACCC-3′

To rescue these infectious cDNA clones *in vitro*, transcription and transfection of the full length viral cDNA clones were performed according to the manufacturer’s instructions. Briefly, the full-length cDNA clone was linearized by cleavage with restriction enzyme *Swa*I, which cuts downstream of the poly (A) tail. Then, the capped RNA transcripts from cDNA were performed using the mMESSAGE MACHINE SP6 kit (Ambion, USA). Subsequently, the *in vitro* transcribed RNA was transfected into BHK-21 cells using DMRIE-C reagent (Invitrogen, USA). To rescue the virus, cell culture supernatant obtained 24 h post-transfection was serially passaged on Marc-145 cells. The infected Marc-145 cells were monitored daily for the formation of CPE (cytopathic effect).

**Figure 3:**
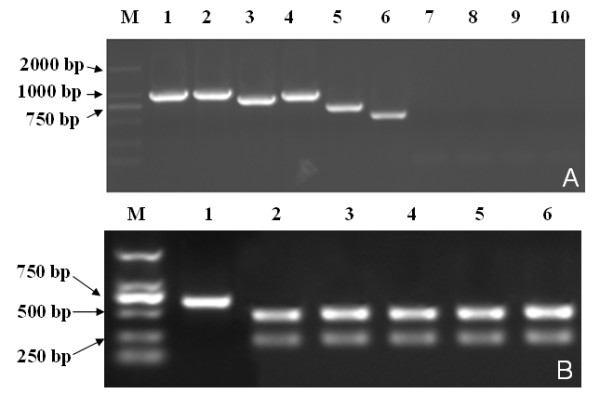
**Identification of the rescued viruses by RT-PCR.** One pair of primers (F2436/R3442) amplifying deletion region of the nsp2 was used to confirm the presence of deletions (**A**). Another pair of primers (R14320/F14752) amplifying part of PRRSV was used to identify the recombinant viruses containing a *Mlu*I enzyme site as gene marker, The results of RT-PCR products digested by *Mlu*I indicated that the recombinant viruses were rescued from cDNA clones (**B**).

**Figure 4:**
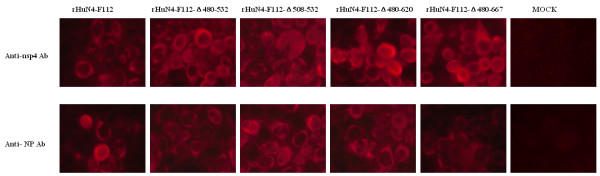
**Identification of the deletion or insertion viruses by IFA test.** Marc-145 cells were infected with the five passages deletion viruses, rHuN4-F112-Δ 480-532, rHuN4-F112-Δ 508-532, rHuN4-F112-Δ 480-620 rHuN4-F112-Δ 480-667 and parental virus rHuN4-F112. Approximately 24 h after infection, cells were processed for indirect immunofluorescence after incubation with Abs specific for PRRSV NP protein (Anti-NP Ab), nsp4 protein (Anti-nsp4 Ab). As expected, the Ab specific for NP protein and nsp4 protein could react with all these 5 recovery viruses.

The successfully recovered recombinant viruses were tested as below. The nsp2 mutant regions of the recombinant viruses were examined by RT-PCR (Reverse Transcriptase Polymerase Chain Reaction) after 3 passages in Marc-145 cells. Two pairs of primers were used to identify each mutant virus: one (F2436/R3442) targeting the deletion region, and the other (F14320/R14752) targeting the mutant *Mlu*I restriction enzyme site. As shown in Figure 3A, all the successfully recovered mutant viruses maintained the respective engineered deletion with the expected size. The mutant *Mlu*I restriction enzyme site of the successfully recovered viruses were tested (Figure 3B), and these regions were also sequenced (at least 10 clones) (data not shown). Subsequently, the successfully recovered viruses were test by immunofluorescence assay (IFA). Marc-145 cells grown on 6-well plates and infected with virus were fixed in 80% cold ethanol at 4°C for 30 min. The cells were incubated for 60 min with PRRSV nucleocapsid protein monoclone antibody and nsp4 protein monoclone antibody, respectively, and then stained with lexa Fluor®568 goat anti-mouse lg G (H + L) (Invitrogen, USA) and examined by fluorescence microscopy. The images were collected under an Olympus IX-70 inverted microscope (Figure 4). The results showed that all the successfully recovered viruses could be detected by antibody of the nsp4 and N protein of PRRSV.

**Figure 5:**
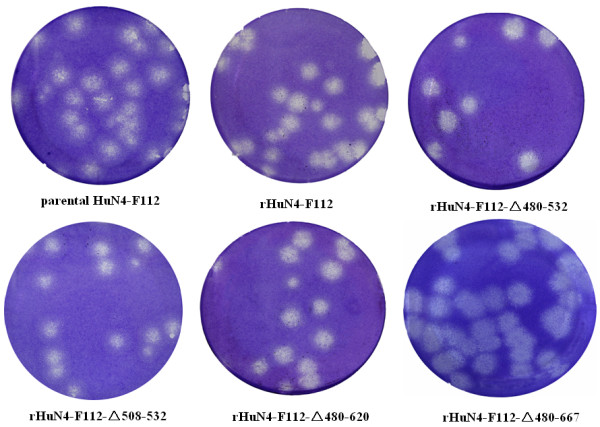
**Plaque assays for parental HuN4-F112, rHuN4-F112, rHuN4-F112-Δ480-532, rHuN4-F112-Δ508-532, rHuN4-F112-Δ480-620, and rHuN4-F112-Δ480-667 were completed in parallel.** MARC-145 cells were infected with viruses, and monolayer was stained with crystal violet at 5 days post infection.

To determine the effect of the nsp2 deletions on PRRSV growth, the ability to form plaques was examined. Confluent monolayers of Marc-145 cells cultured in a 6-well plate were infected with 10-fold serially diluted viruses (10 to 0.001 TCID_50_). After 1 h incubation, the excessive inoculum was removed and an agar overlay was applied onto the monolayer. Plaques were stained at 37 °C with crystal violet 5 days post infection. The results showed that the plaque sizes of the deletion viruses had no significant difference in comparison to that of parental virus HuN4-F112 (Figure 5).

**Figure 6:**
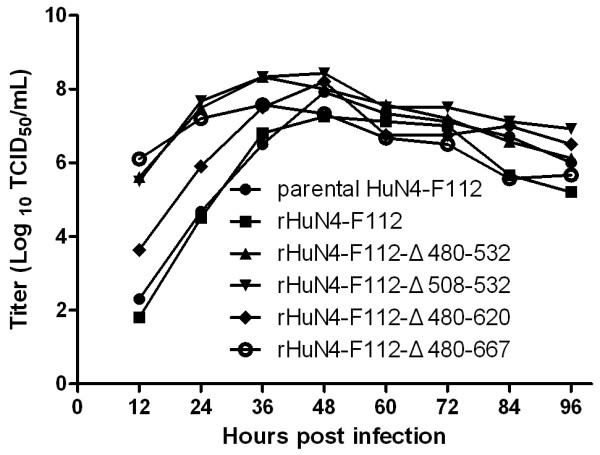
**Viral growth curve analysis. Marc-145 cells in T-25 flasks were infected with PRRSV nsp2 small deletion mutants and parental virus at 100TCID**_**50**_**.** The cell supernatants were collected for titration analysis by the method of Reed-Muench at every 12 h hpi. The virus was titrated on Marc-145 cells.

Viral growth assays was finished as the papers reported. The titers of PRRSV were all expressed as TCID_50_. Marc-145 cell monolayer in T-25 flasks was infected with either parental or mutant viruses at 100TCID_50_. After 1 h of attachment at 37°C in 5% CO_2_ with gentle mixing every 15 minutes, the inocula were removed and the monolayer was washed three times with serum-free DMEM. After washing, 5 ml 2% FBS DMEM was added and the flasks were then incubated for up to 4 days at 37°C in 5% CO_2_. Samples were collected from the medium at every 12 hours post infection (hpi) and titrated by the method of Reed-Muench on Marc-145 cells in 96-well plates. The results of the viral growth curve analysis (Figure 6) showed that nsp2 with small deletions did not affect the viral growth on Marc-145 cells, but the titers of the deletion virus were higher than the parental virus during the early stage of infection (before 36 h). The titers of the rHuN4-F112-Δ480-532, rHuN4-F112-Δ508-532, and rHuN4- F112-Δ480-667 before 36 h were about 1000-fold higher than that of the parental virus HuN4-F112, and the titers of the rHuN4-F112-Δ480-620 before 36 h were about 100-fold higher than that of the parental virus HuN4-F112.

In this study, eight deletion constructs were produced as shown in Figure 2: rHuN4-F112-Δ480-532, rHuN4-F112-Δ508-532, rHuN4-F112-Δ480-620, rHuN4- F112-Δ480-667, rHuN4-F112-Δ420-532, rHuN4-F112-Δ400-532, rHuN4-F112- Δ444-667, and rHuN4-F112-Δ400-700. The transfection results suggested that four mutants (rHuN4-F112-Δ480-532, rHuN4-F112-Δ508-532, rHuN4-F112-Δ480-620, and rHuN4-F112-Δ480-667) resulted in viable viruses. All others produced nonreplicating PRRSV genomes, and the deletion cDNA clones appeared to be lethal to the virus, which have been rescued at least five clones. Recently, Han J *et al.* reported that deletion aa 324 to 746 of nsp2 of PRRSV VR-2332 strain did not affect the viral replication, and they also found that the mutant virus displayed decreased cytolytic activity on Marc-145 cell and did not develop visible plaques [15]. The combined data showed that HuN4-F112 nsp2 harbors smaller nonessential region for viral replication than VR-2332 strain in cell culture. Nsp2 between the HuN4-F112 and VR-2332 have different length and share less than 70% similarity of their amino acid, and this may be the reason why the nonessential regions is different among the different PRRSV strains.

Although deletions of the segments of nsp2 [amino acids (aa) 480 to 667] did not affect the viral growth on Marc-145 cells, deletion of these regions led to earlier PRRSV replication increased (before 36 h after infectious *in vitro*), which suggested that the sequences of the nonessential regions of nsp2 might play an important role in regulating the viral replication through an unknown mechanism. Recently, nsp1, nsp2, nsp4 and nsp11 were found to have strong ability to moderate inhibitory effects on beta interferon (IFN-β) promoter activation to regulate host innate immune response, but the roles of the nsp4 were not clear [28]. Therefore, the function of these nonessential sequences of nsp2 should be under investigated.

PRRSV as a potential viral vector has been investigated previously. Most of the concern was focused on the region between the ORFs and the 3′ end of the genome [29-31], while recently some researchers were interested in using nsp2 to express foreign gene since the nonessential regions of nsp2 for replication had been identified [15,32-34]. When viral structural proteins were used to express the foreign genes, the size of the insertion was limited to less than 10 amino acids [29]. Inserting the fragment into the internal ORFs would affect the PRRSV replication. Therefore, the nonessential region of PRRSV nsp2 suggested the likelihood of expressing a large foreign gene as a fusion protein in the viral replicase region. Therefore, these identified nonessential regions suggested they might be the potential inserted sites to express the foreign gene in PRRSV genome.

## Competing interests

The authors declare that they have no competing interests.

## Authors’ contributions

YZX and GZT conceived the study and wrote the paper. YZX, YJZ, SRZ, WT, LL, and YFJ performed the experiments. YZX analyzed the data. All authors have read and approved the final manuscript.
